# Nitric oxide targets oligodendrocytes and promotes their morphological differentiation

**DOI:** 10.1002/glia.22759

**Published:** 2014-10-18

**Authors:** Giti Garthwaite, Kathryn Hampden-Smith, Gary W Wilson, David A Goodwin, John Garthwaite

**Affiliations:** From the Wolfson Institute for Biomedical Research, University College LondonLondon, WC1E 6BT, United Kingdom

**Keywords:** cGMP, myelination, cerebellum, brainstem, optic nerve, natriuretic peptide

## Abstract

In the central nervous system, nitric oxide (NO) transmits signals from one neurone to another, or from neurones to astrocytes or blood vessels, but the possibility of oligodendrocytes being physiological NO targets has been largely ignored. By exploiting immunocytochemistry for cGMP, the second messenger generated on activation of NO receptors, oligodendrocytes were found to respond to both exogenous and endogenous NO in cerebellar slices from rats aged 8 days to adulthood. Atrial natriuretic peptide, which acts on membrane-associated guanylyl cyclase-coupled receptors, also raised oligodendrocyte cGMP in cerebellar slices. The main endogenous source of NO accessing oligodendrocytes appeared to be the neuronal NO synthase isoform, which was active even under basal conditions and in a manner that was independent of glutamate receptors. Oligodendrocytes in brainstem slices were also shown to be potential NO targets. In contrast, in the optic nerve, oligodendrocyte cGMP was raised by natriuretic peptides but not NO. When cultures of cerebral cortex were continuously exposed to low NO concentrations (estimated as 40–90 pM), oligodendrocytes responded with a striking increase in arborization. This stimulation of oligodendrocyte growth could be replicated by low concentrations of 8-bromo-cGMP (maximum effect at 1 µM). It is concluded that oligodendrocytes are probably widespread targets for physiological NO (or natriuretic peptide) signals, with the resulting rise in cGMP serving to enhance their growth and maturation. NO might help coordinate the myelination of axons to the ongoing level of neuronal activity during development and could potentially contribute to adaptive changes in myelination in the adult.

## Introduction

Nitric oxide (NO) is a unique, membrane-permeating type of transmitter that operates throughout the central nervous system. At this location, NO is generated largely by the neuronal subtype of NO synthase (nNOS) in response to a rise in intracellular Ca^2+^, typically brought about through NMDA receptor channels, and acts in target cells by stimulating specialized guanylyl cyclase-coupled receptors to generate cGMP (Garthwaite, 2008; Hardingham et al., 2013; Steinert et al., 2010). NO from endothelial NO synthase (eNOS) may also be important for brain function independently of its role in the control of blood flow in that it has been shown to regulate the membrane potential of axons in the optic nerve (Garthwaite et al., 2006) and to participate in synaptic plasticity in the hippocampus (Hopper and Garthwaite, 2006; Son et al., 1996; Wilson et al., 1999) as well as in various behaviors (Demas et al., 1999). The main established targets of endogenously-produced NO in the central nervous system are neurones and astrocytes and most of the relevant literature to date has dwelt on these cell types, although neuronally-derived NO may also act on the microvasculature to regulate blood flow (Yang et al., 2003).

The possible action of NO on oligodendrocytes has so far been almost entirely confined to its putative pathological role in the context of multiple sclerosis (Lassmann, 2011; Smith and Lassmann, 2002). Such deleterious effects are likely to involve NO in high concentrations inhibiting mitochondrial respiration and contributing to oxidative/nitrosative stress through peroxynitrite formation. Nonetheless, there is evidence that NO contributes to the increased phosphorylation of myelin basic protein in oligodendrocytes taking place after high-frequency stimulation of hippocampal axons (Atkins and Sweatt, 1999). There is also one report that, based on cGMP immunohistochemistry, NO induces cGMP accumulation in oligodendrocytes in the corpus callosum in incubated brain slices from the 2-week-old rat and that this response diminishes progressively with age thereafter (Tanaka et al., 1997). This finding points to a potential influence of NO on oligodendrocyte development. In contrast, no cGMP accumulation in oligodendrocytes in slices of the developing (8-day-old) rat hippocampus following NO administration was found (Bartus et al., 2013). Similarly, in the optic nerve of 10-day-old (Garthwaite et al., 2006) or adult (Garthwaite et al., 1999) rats, NO-evoked cGMP accumulation was prominent in axons but was not detected in oligodendrocytes.

One of the best-studied brain areas with respect to NO-cGMP signaling is the cerebellum, an area important for motor control and one in which this pathway contributes to synaptic plasticity and other phenomena (Contestabile, 2012; Garthwaite, 2008). cGMP immunohistochemistry and attempts to locate NO-activated guanylyl cyclase subunits by *in situ* hybridization or immunochemistry in the rodent cerebellum identify neurones and astrocytes as the main NO targets (de Vente et al., 1990; Ding et al., 2004; Furuyama et al., 1993; Gibb and Garthwaite, 2001; Southam et al., 1992) but no attempt appears to have been made to investigate oligodendrocytes, either during development or in adulthood. Following exposure of slices of the developing cerebellum to exogenous NO, abundant cells in the white matter were shown to accumulate cGMP but these cells were considered to be astrocytes (de Vente et al., 1990).

In view of the questionable status of oligodendrocytes as physiological targets for NO, we have examined the ability of this cell type to accumulate cGMP in response to exogenous and endogenous NO, using cGMP immunohistochemistry and slices of rat cerebellum at different developmental stages. Natriuretic peptides, such as atrial natriuretic peptide (ANP), whose receptors also possess intrinsic guanylyl cyclase activity (Cao and Yang, 2008), were examined as alternative agonists for cGMP generation by oligodendrocytes. The positive outcome of these studies spawned tests of the possibility that the NO-cGMP pathway influences oligodendrocyte development.

## Materials and Methods

### Animals

This study used male Sprague-Dawley rats aged 3–63 days and 11–14 day-old wild-type mice and mice deficient in endothelial NO synthase (eNOS^−/−^), the day of birth being called day 1. Homozygous eNOS^−/−^ mice (SV129 and C57BLK/6 background) were bred in-house (donated by Dr. Adrian Hobbs). The wild-type mice were SV129-C57BLK/6 F1 hybrids (Harlan UK Limited, Bicester, UK). Timed pregnant mice used for tissue culture were the CD-1 strain (Charles River) and were donated by Prof. William D. Richardson. All animal use was approved by the local (UCL) ethics committee and was carried out strictly in accordance with the UK Animals (Scientific Procedures) Act 1986.

### Special Chemicals

1-Benzyl-3-(hydroxymethyl-2-furyl)indazole (YC-1), diethylammonium (Z)-1-(N,N-diethylamino)diazen-1-ium-1,2-diolate (DEA/NO), (*Z*)-1-[*N*-(3-ammoniopropyl)-*N*-(n-propyl)-amino]/NO (PAPA/NO), (*Z*)-1-[2-(2-aminoethyl)-*N*-(2-ammonioethyl)amino]diazen-1-ium-1,2-diolate (DETA/NO) and 5-cyclopropyl-2-{1-(2-fluorobenzyl)-1*H*-pyrazolo[3,4-b]pyridin-3-yl}pyrimidin-4-ylamine (BAY41-2272), were from Enzo Life Sciences (Exeter, UK); stock solutions of NO donors were made up in 10 mM NaOH and kept on ice. ANP, C-type natriuretic peptide (CNP), 8-Bromo-cGMP (8-Br-cGMP), bicuculline, and isobutylmethylxanthine (IBMX) were from Sigma-Aldrich (Dorset, UK); 1*H*-[1,2,4], oxadiazolo[4,3-a]quinoxalin-1-one (ODQ), L-nitroarginine (L-NNA)), N-methyl-D-aspartate (NMDA), 6-cyano-7-nitroquinoxaline-2,3-dione (CNQX), 2-amino-5-phosphonopentanoic acid (AP5), and 2-[(1*S*,2*S*)-2-carboxycyclopropyl]-3-(9*H*-xanthen-9-yl)-D-alanine (LY341495) were from Tocris Cookson (Bristol, UK); tetrodotoxin (TTX) was from Latoxan Laboratories (Rosans, France); Vectastain elite ABC KIT was from Vecto Labs Ltd (Peterborough, UK); 3,3′-diaminobenzidine (DAB) was from Sigma-Aldrich (Dorset, UK); Mayer's haemalum was from Raymond A Lamb Ltd (Eastbourne UK). Common laboratory chemicals were from Sigma-Aldrich and BDH Merck Ltd (Poole, Dorset, UK).

### Brain Slices and Optic Nerves

Brain slice methods were as used previously (Southam et al., 1991). Briefly, 400 µm thick sagittal cerebellar slices from 4- to 14-day-old animals were cut using a McIlwain tissue chopper whereas a vibroslicer (Intracel 1000 plus; Intracel Ltd., Royston, UK) was used to prepare 300 µm thick slices from older animals. Coronal slices of brainstem (300 µm thickness) were cut at the level of 7th nerve from 14-day-old rats using the vibroslicer. Optic nerves from rats and mice were isolated and incubated *in vitro* as described (Garthwaite et al., 2006). Brain slices or optic nerves from different animals were randomized and allowed to recover for at least 1 h in artificial CSF (aCSF) solution in flasks held in a shaking water bath at 37°C, before experiments began. The aCSF was composed of (mM): NaCl (120) KCl (2), CaCl_2_ (2), NaHCO_3_ (26) KH_2_PO_4_ (1.18), MgSO_4_ (1.19), and glucose (11), continuously gassed with 95% O_2_ and 5% CO_2_. The Ca^2+^-free aCSF had a raised MgSO_4_ concentration (4.2 mM) and was supplemented with EGTA (0.2 mM). Most experiments were carried out in the presence of the general phosphodiesterase inhibitor IBMX (1 mM; 10 min preincubation) and, when used, antagonists were added 10 min prior to IBMX. Following treatment, brain slices or optic nerves were either fixed for immunohistochemistry or were inactivated in boiling tris-HCl buffer (50 mM, pH 7.5) containing EDTA (4 mM) and homogenized by sonication after which aliquots were removed for measurement of protein (bicinchoninic acid method) and cGMP (radioimmunoassay).

### Immunohistochemistry

cGMP immunohistochemistry was performed on sister tissues to those used for cGMP measurement. The specimens were fixed in ice-cold, freshly-depolymerized paraformaldehyde (4%) in 0.1 M phosphate buffer (pH 7.4) for 2 h and usually then processed for frozen (10 µm) sectioning, cerebellar slices being cut in the sagittal plane, brainstem slices in the coronal plane, and optic nerves, longitudinally. Some optic nerves were processed for semi-thin sectioning, for which they were embedded in Durcupan resin in such a way that half of each nerve could be sectioned (1 µm) longitudinally and the other half transversely (Garthwaite et al., 1999). Immunostaining of frozen tissues was performed generally as described (Garthwaite et al., 2006). Briefly, sections were brought to room temperature, incubated for 10 min with tris-buffered saline containing 0.1% triton-X-100 (TBST), pH 7.6, at room temperature, and then blocked for 1 h with 10% normal donkey serum in TBST at 4°C. The sections were then incubated with a mixture of primary antibodies in TBST overnight at 4°C. After washing in TBST (3 × 5 min), sections were incubated with appropriate secondary antibodies at room temperature for 1 h, washed (3 × 5 min) in TBST before being mounted in Vectashield mounting medium containing the nuclear stain 4',6-diamidino-2-phenylindole (DAPI; Vector Laboratories, Peterborough, UK). In some experiments, TO-PRO-3 (1:50,000, 5 min; Invitrogen, Paisley, UK) was used instead of DAPI. Resin-embedded, semi-thin sections were immunostained as before (Garthwaite et al., 1999). Briefly, the sections were etched with a 1:1 mixture of ethanol and saturated sodium hydroxide in ethanol for 5 min at room temperature prior to incubation with primary antibodies against cGMP and CNPase (as below). Immunoperoxidase staining was carried out using rabbit biotinylated anti-sheep antibody (Vector Laboratories; 1:1000, 1 h) followed by Vector stain ABC elite (30 min) and then 3,3′-diaminobenzidine (4 min) followed by counterstaining in Mayer's haemalum (5 min).

For nNOS and eNOS immunohistochemistry, the procedure was as described above except that the tissues were fixed in 1% paraformaldehyde. As controls for all the immunohistochemistry, the primary antibodies were omitted.

The primary antibodies and their sources were: sheep anti-cGMP (a gift from Dr Jan de Vente, Maastricht, Netherlands; 1:1000); monoclonal mouse anti-2′,3′-cyclic nucleotide 3′-phosphodiesterase (CNPase; 1:2000) and monoclonal mouse anti-myelin oligodendrocyte glycoprotein (MOG; 1:200), both from Millipore (Watford, UK); mouse monoclonal anti-myelin basic protein (MBP; Serotec, Herts, UK; 1:100); polyclonal rabbit anti-glial fibrillary acidic protein (GFAP; Sigma-Aldrich; 1:1000), polyclonal rabbit anti-neuronal NO synthase (Zymed, San Francisco, CA, USA; 1:500), monoclonal mouse anti-endothelial NO synthase (BD Transduction Laboratories, Lexington, KY, USA; 1:200); polyclonal rabbit anti-neurofilament-68 (NF68; 1:1000), anti-neurofilament-200 (NF200; 1:1000), anti-NG2 (1:500), and anti-ANP (1:100) all from Chemicon Europe (Hampshire, UK); rabbit anti-CNP peptide-22 (1:200) from Phoenix Pharmaceuticals Inc., Burlingame, CA, USA. Secondary antibodies were: Alexa Fluor 488 donkey anti-sheep IgG (1:1000), donkey anti-rabbit IgG (1:1000) and donkey anti-mouse IgG (1:200); Alexa Fluor 568 donkey anti-mouse IgG (1:500) and donkey anti-rabbit IgG (1:200); Alexa Fluor 594 donkey anti-mouse IgG (1:500), donkey anti-rabbit (1:1000) and donkey anti-rat IgG (1:500), all from Invitrogen; fluorescein-labeled donkey anti-sheep and rhodamine-labeled donkey anti-mouse (1:200), both from Chemicon. Images were captured in a confocal microscope (TCS-DMRE-7 or SP1-DMRE-7, Leica, Milton Keynes, UK). The titres of secondary antibodies were chosen from an initial series of double dilutions within the range recommended by the manufacturer; lack of bleed-through was verified using single-labeled sections.

### Primary Cultures

Cultures from mouse E14.5 cerebral cortices were prepared using a published method (Tekki-Kessaris et al., 2001) with minor modifications. Briefly, the cortices were dissected into ice-cold Earle's buffered saline solution (Gibco BRL, Paisley, UK) without Ca^2+^ or Mg^2+^ and then incubated in Earle's buffered saline solution containing trypsin (0.00625% w/v) and DNase I (13 µg/mL) at 37°C for 40–50 min. Digestion was halted by the addition of Dulbecco's modified Eagle's medium (DMEM) containing 10% fetal bovine serum and cells dissociated by tapping sharply on the bench 4–5 times. The medium was removed and saved and the tapping process repeated. The pooled media were centrifuged and the cells resuspended in a defined medium comprising DMEM supplemented with transferrin (100 µg/mL), bovine serum albumin (100 µg/mL), progesterone (20 nM), sodium selenite (30 nM), thyroxine (400 ng/mL), triido-L-thyronine (300 ng/mL), putrescine (100 µM), insulin (5 µg/mL), and human basic fibroblast growth factor (bFGF; 20 ng/mL) which stimulates the generation of oligodendrocyte progenitor cells (Kessaris et al., 2004). The cells were then plated onto glass coverslips pre-coated with poly-D-lysine (0.02 mg/mL) at a density of 1.25 × 10^5^ cells (0.35 mL) per well and incubated at 37°C in a 5% CO_2_ incubator. Half of the medium was changed every other day and bFGF was removed on day 3 post-plating. The development of oligodendrocytes in the cultures was initially characterized by fixation (ice-cold 4% paraformaldehyde for 40 min) followed by immunolabeling for NG2, CNPase, MBP, and MOG. Selected cultures were also immunostained for astrocytes (GFAP), nNOS, and cGMP.

### Image Analysis

For quantifying colocalization of cGMP with CNPase, MOG or NF68 in cerebellar slices and optic nerve, confocal *z*-stacks (0.75–1 µm) were acquired with the gain and offset being kept constant. JACoP (Just Another Colocalisation Plugin) was used in ImageJ to calculate the Manders' M1 overlap coefficient on the average projection image generated from the *z-*stack (http://rsbweb.nih.gov/ij/index.html). Colocalization of cGMP and CNPase in cortical cultures was analyzed from z-stacks (1 µm apart) of fixed cells in ImageJ. Binary images were first created using a set threshold (which remained the same for all images analyzed) and they were then combined using the AND logic operator; the histogram function was used to calculate the number of coinciding pixels that were above the threshold in both red and green channels (Bolte and Cordelieres, 2006).

To analyze changes in oligodendrocyte morphology in cortical cultures, confocal images at low magnification (16X) were captured from five fields (each measuring 1 mm^2^) per coverslip. The fields were chosen as follows: imaginary axes were drawn through the centre of the coverslip at right angles to each other. The first image was taken from the centre and the other four were two fields away on each axis. The images were transferred to a computer and quantified in a blinded manner by two independent observers. The areas occupied by the oligodendrocyte processes were demarcated with circles and the cells arbitrarily divided into two groups having overall diameters below and above 70 µm. The percentage of cells with diameter above 70 µm was then calculated for each experimental condition. In selected conditions, the exact diameters of oligodendrocytes wider than 70 µm were measured in five areas of each coverslip, selected as described above.

To quantify oligodendrocyte cGMP accumulation in optic nerves from 7-day-old rats, confocal images were taken from the middle and 100 µm from each end (chiasmatic and global) of each of three longitudinal sections per nerve (100 µm or more apart). Counts of total CNPase-positive cell bodies with and without cGMP staining were performed on multiple 400 × 400 µm fields in a blinded fashion by two independent observers. Only those CNPase-stained cells showing complete cytoplasmic fluorescence were scored as CNPase-positive cells.

### Modeling NO Concentrations

NO concentrations in the tissue-culture medium resulting from release of NO from DETA/NO were calculated by solving the following pair of differential equations in Mathcad 14 (Parametric Technology Corp., Needham, MA, USA):


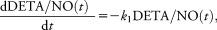



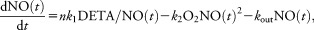


where *k*_1_ = rate constant for DETA/NO decomposition (9.627 × 10^−6^ s^−1^ for a 20 h half-life), *k*_2_ = autoxidation rate constant (1.36 × 10^7^ M^−2^ s^−1^; Schmidt et al., 1997), *n* is the stoichiometry of NO release from DETA/NO (assumed to be 2; Mooradian et al., 1995), *O*_2_ = oxygen concentration (0.21 mM for an air-equilibrated solution), and *k*_out_ is the rate constant for loss of NO by mass transfer into the atmosphere. When NO is generated within a tissue-culture dish, the atmosphere immediately above the medium is likely to be effectively NO-free and mass transfer into this phase will represent a significant loss of NO from the medium during longer experiments (Chen and Deen, 2001). Having limited water solubility, mass transfer of NO will be governed almost entirely by its rate of exit from the aqueous phase, so that *k*_out_ = *k_L_*(*A/V*), where *k_L_* is the liquid mass transfer coefficient, which was taken to be the value for O_2_ mid-way between values for minimally-agitated samples of distilled water and sea-water saline at 37°C, that is, 5.6 × 10^−4^ cm s^−1^ (Downing and Truesdale, 1955); *A* = area of medium exposed to the atmosphere (1.327 cm^2^ for a 1.3 cm diameter tissue-culture well) and *V* = volume of medium (0.35 cm^3^). Using these values, *k*_out_ = 2.124 × 10^−3^ s^−1^. Cellular mechanisms that consume NO may also be present (Griffiths and Garthwaite, 2001), so the NO concentrations calculated in this way could be overestimates.

### Statistics

Values are presented as means ± SEM; statistical analysis used one-way ANOVA; a *P* value of <0.05 was considered significant.

## Results

The general strategy initially was to examine the cellular location of cGMP using immunocytochemistry in and around the white matter of cerebellar slices after stimulation with exogenous or endogenous NO. Exogenous NO was usually supplied by PAPA/NO at a concentration (30 µM) giving half-maximal or maximal cGMP accumulation in rat optic nerve depending on age (see below). To discern the targets of endogenous NO, the slices were exposed to BAY 41-2272 or YC-1, these being allosteric enhancers of NO-activated guanylyl cyclase (Evgenov et al., 2006; Roy et al., 2008) able to amplify endogenous NO-stimulated cGMP accumulation into the range that is detectable immunocytochemically (Bartus et al., 2013; Garthwaite et al., 2006). The other main stimuli applied to the slices were ANP, which activates a transmembrane guanylyl cyclase-coupled receptor (Cao and Yang, 2008), and the glutamate agonist NMDA whose receptors, when stimulated, couple to nNOS activation (Garthwaite, 2008). The NMDA concentration chosen (30 µM) gives approximately half-maximal cGMP accumulation in cerebellar slices from the developing rat (Garthwaite, 1985) and the ANP concentration (1 µM) yields a maximal cGMP response from adult guinea-pig cerebellar slices (Hernandez et al., 1994) or rat optic nerve (see below). Immunostaining for CNPase was routinely used to identify oligodendrocytes.

### cGMP Accumulation in Oligodendrocytes During Cerebellar Development

**Figure 1:**
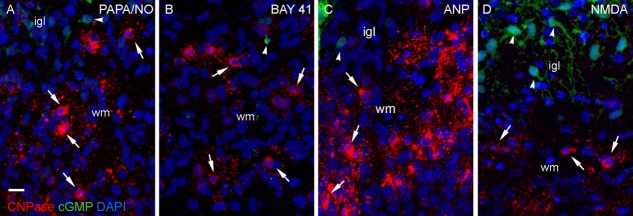
Lack of oligodendrocyte cGMP immunostaining in 3-day-old rat cerebellar slices. Sections show the primitive white matter (wm) in slices previously exposed to (A) PAPA/NO (30 μM, 5 min), (B) BAY 41-2272 (3 μM, 5 min), (C) ANP (1 μM, 10 min), and (D) NMDA (30 μM, 2 min), all in the presence of IBMX (1 mM). Sections were stained for CNPase (red), cGMP (green) and nuclei (DAPI, blue) and the images overlayed. Key: arrows, CNPase-positive oligodendrocytes; arrowheads, putative astrocytes; igl, internal granule cell layer. Images are representative of 4–5 slices in two experiments. Scale bar (A) =13 μm. [Color figure can be viewed in the online issue, which is available at http://wileyonlinelibrary.com.]

In slices of 3-day-old rat cerebellum incubated with the general phosphodiesterase inhibitor IBMX (1 mM), PAPA/NO, BAY 41-2272, ANP, and NMDA all evoked cGMP immunostaining of putative astrocytes but CNPase-positive oligodendrocytes were unstained (Fig. 1A–D).

**Figure 2:**
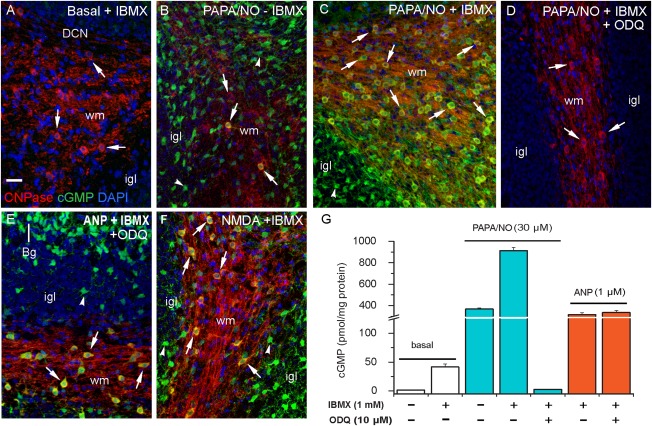
NO and ANP stimulate cGMP accumulation in oligodendrocytes in 8- to 9-day-old rat cerebellar slices. The immunohistochemical images (A–F) are all overlayed (see A for color code; yellow indicates coincident cGMP and CNPase staining). Conditions are given at the top of each panel (PAPA/NO, 30 μM, 5 min; ANP, 1 μM, 10 min; NMDA, 30 μM, 2 min). When present, ODQ and IBMX concentrations were at 10 μM and 1 mM, respectively. Key: DCN, deep cerebellar nucleus; Bg, Bergmann glia; igl, internal granule cell layer; wm, white matter; arrows, oligodendrocytes; arrowheads, putative astrocytes. Pictures are representative of 6–8 slices from 2–3 different experiments. Scale bar (in A) = 25 μm (applies to all panels). (G) cGMP levels in slices measured in parallel by radioimmunoassay (*n* = 6–12 from 2–3 separate experiments). [Color figure can be viewed in the online issue, which is available at http://wileyonlinelibrary.com.]

**Figure 3:**
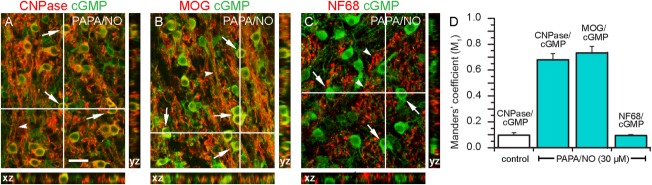
Colocalization analysis of cGMP immunoreactivity in the white matter of 10-day-old rat cerebellar slices. The slices were exposed to PAPA/NO (30 μM, 5 min) in the presence of IBMX (1 mM) and sections stained for cGMP together with CNPase, MOG, or NF68. (A–C) overlayed images of the x-y and corresponding orthogonal projections (z) at the level of the cross-hairs. Merged structures appear yellow. The images were prepared from confocal z-stacks taken at 1 μm intervals. The results are representative of three slices in two different experiments. Arrows, oligodendrocytes; arrowheads, axons in longitudinal (A, B) and cross-sectional (C) profiles. Scale bar (A) = 25 μm (applies to all images). The histogram (D) shows mean values (±SEM) of the Manders' M_1_ colocalization coefficient determined from an area of 0.32 mm^2^ in each section; a value of 1.0 signifies 100% colocalization. [Color figure can be viewed in the online issue, which is available at http://wileyonlinelibrary.com.]

With tissue from 8- to 10-day-old animals, little or no cGMP immunolabeling was apparent under basal conditions, when only IBMX (1 mM) was present (Fig. 2A). With the further addition of PAPA/NO, cGMP immunoreactivity was seen over the majority of oligodendrocytes (Fig. 2C). This response was inhibited by 10 µM ODQ (Fig. 2D), a compound that blocks the activation of guanylyl cyclase by NO (Garthwaite et al., 1995). To test more rigorously for colocalization, the tissue was immunostained for cGMP along with CNPase, or MOG, a marker for mature oligodendrocytes, or NF68, an axonal marker. The orthogonal projections showed colocalized immunostaining between cGMP, CNPase, and MOG, but not between cGMP and NF68 (Fig. 3A–C). The conclusion from these results, namely that cGMP immunostaining was in oligodendrocytes and not in axons, was verified by a colocalization analysis (Fig. 3D).

Without IBMX, only very few oligodendrocytes generated detectable cGMP immunostaining in response to PAPA/NO (Fig. 2B), suggesting that phosphodiesterase enzymes actively suppress NO-stimulated cGMP accumulation in this cell type. By contrast, putative astrocytes were cGMP-positive with or without IBMX (Fig. 2B,C), consistent with cerebellar astrocytes having only low cGMP-hydrolyzing phosphodiesterase activity (Bellamy and Garthwaite, 2001). With IBMX included, ANP (in the presence of ODQ) also caused extensive cGMP accumulation in oligodendrocytes (Fig. 2E). Moreover, NMDA provoked robust oligodendrocyte cGMP accumulation, but only in those regions of the white matter adjacent to the internal granule cell layer (Fig. 2F), this being a layer rich in neurones expressing nNOS and NMDA receptors (Contestabile, 2012; Moriyoshi et al., 1991). Measurements made in three sections (two experiments) in which the white matter was sufficiently wide (>100 µm) indicated that the cGMP immunostaining diminished progressively with distance from the internal granule cell layer into the white matter, to be just detectable 30 ± 4 µm away.

In parallel, cGMP in the slices was measured directly by radioimmunoassay (Fig. 2G). Inclusion of IBMX raised cGMP levels from near zero to approximately 40 pmol/mg protein although this condition gave only faint immunostaining of some putative astrocytes (Fig. 2A). PAPA/NO elevated cGMP much further, to about 400 pmol/mg protein. The response to PAPA/NO was more than doubled in the presence of IBMX and was inhibited almost completely by ODQ. ANP (in the presence of IBMX) also elevated cGMP to high levels (about 300 pmol/mg protein) both in the absence and presence of ODQ (Fig. 2G).

**Figure 4:**
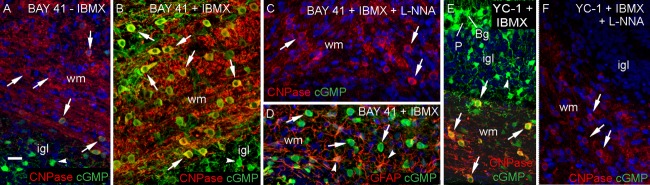
Endogenous NO targets oligodendrocytes in 8- to 9-day-old rat cerebellar slices. The slices were exposed for 5 min to BAY 41-2272 (3 μM, 5 min; A–D) or YC-1 (50 μM, 5 min; E, F) in the absence (A) or presence (B–F) of IBMX (1 mM). Cryostat sections were immunostained for cGMP (green) together with CNPase (red; A–C, E, F) or GFAP (red; D). Images are all overlays in which green/red co-staining appears yellow. Nuclei are stained with DAPI (blue). The cGMP immunostaining was abolished by L-nitroarginine (L-NNA, 100 μM; C, F). Key: arrows, oligodendrocytes; arrowheads, GFAP-positive astrocytes (D) and putative astrocytes (A, B, E); wm, white matter; igl, internal granule cell layer; P, Purkinje cell; Bg, Bergmann glia. Images are representative of 4–6 slices from two experiments. Scale bar (A) = 25 μm (applies to all panels). [Color figure can be viewed in the online issue, which is available at http://wileyonlinelibrary.com.]

To determine if these findings using exogenous NO are relevant to endogenous NO signaling, slices were exposed to the allosteric enhancers of NO-activated guanylyl cyclase, BAY 41-2272 (3 µM) and YC-1 (50 µM). In the absence of IBMX, incubation with BAY 41-2272 led to marked cGMP immunostaining of putative astrocytes but only occasionally of oligodendrocytes (Fig. 4A). With IBMX present, almost all oligodendrocytes accumulated cGMP (Fig. 4B), and this response was abolished by the NO synthase inhibitor, L-nitroarginine (Fig. 4C). Some (but not all) GFAP-positive astrocytes in the white matter were also cGMP-positive following incubation with IBMX plus BAY 41-2272 (Fig. 4D). Similarly to BAY 41-2272, the weaker allosteric enhancer YC-1 also stimulated oligodendrocyte cGMP accumulation (Fig. 4E) and this response was eliminated by L-nitroarginine (Fig. 4F). The corresponding cerebellar slice cGMP measurements are included in a later figure (see Fig. 8D).

**Figure 5:**
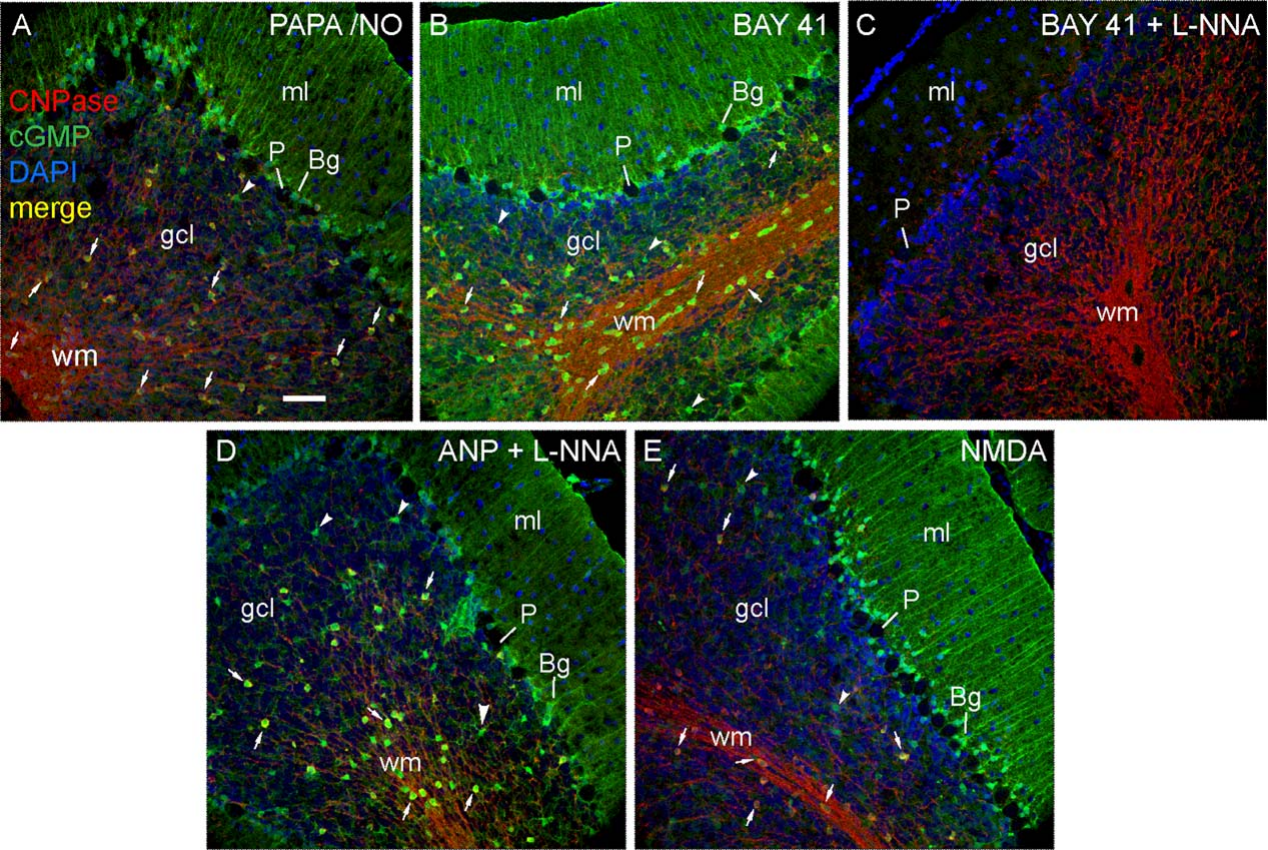
Targeting of oligodendrocytes in slices of adult rat cerebellum by exogenous and endogenous NO. Slices were stimulated by (A) PAPA/NO (30 μM, 5 min), (B, C) BAY 41-2272 (3 μM, 5 min), (D) ANP (1 μM, 10 min) or (E) NMDA (30 μM, 2 min) all in the presence of IBMX (1 mM). L-nitroarginine (L-NNA, 100 μM) was present in C and D. Sections were stained for cGMP (green), CNPase (red) and DAPI (blue). Images are all overlays (red/green co-staining appears yellow) and are representative of six slices per condition in two experiments. Key: ml, molecular layer; P, Purkinje cell; Bg, Bergmann glia; gcl, granule cell layer, wm = white matter; arrows, oligodendrocytes; arrowheads, putative astrocytes. Scale bar (A) = 50 μm (applies to all panels). [Color figure can be viewed in the online issue, which is available at http://wileyonlinelibrary.com.]

The only previous study of this type had indicated that corpus callosal oligodendrocyte cGMP accumulation in response to NO is only transient during development, being most evident in the 2-week-old rat and declining thereafter (Tanaka et al., 1997). To examine the applicability of this result to the cerebellum, oligodendrocytes in slices from 3-week-old and adult (6-weeks-old) rats were tested for sensitivity to exogenous and endogenous NO, as well as to ANP. The adult tissue responded similarly to the slices from immature (8- to 10-day-old) animals described above. In brief, exposure to PAPA/NO caused cGMP immunostaining of oligodendrocytes located in the white matter and also within the internal granule cell layer (Fig. 5A). BAY 41-2272 generated a similar profile of cGMP immunostaining (Fig. 5B) that was abolished by L-nitroarginine (Fig. 5C). ANP (in the presence of L-nitroarginine) also evoked cGMP accumulation in oligodendrocytes in white and grey matter (Fig. 5D), as did NMDA (Fig. 5E). Strong cGMP immunoreactivity in Bergmann glia and other cells presumed to be astrocytes was also observed with PAPA/NO, BAY 41-2272 (without L-nitroarginine), ANP, and NMDA. Enlargements from Fig. 5 are in Supporting Information Fig. S1. The results using 3-week-old rat cerebellar slices subjected to PAPA/NO were closely comparable to those from the adult (not illustrated).

### Sites of NO-Stimulated cGMP Accumulation in Rat Brainstem Slices

**Figure 6:**
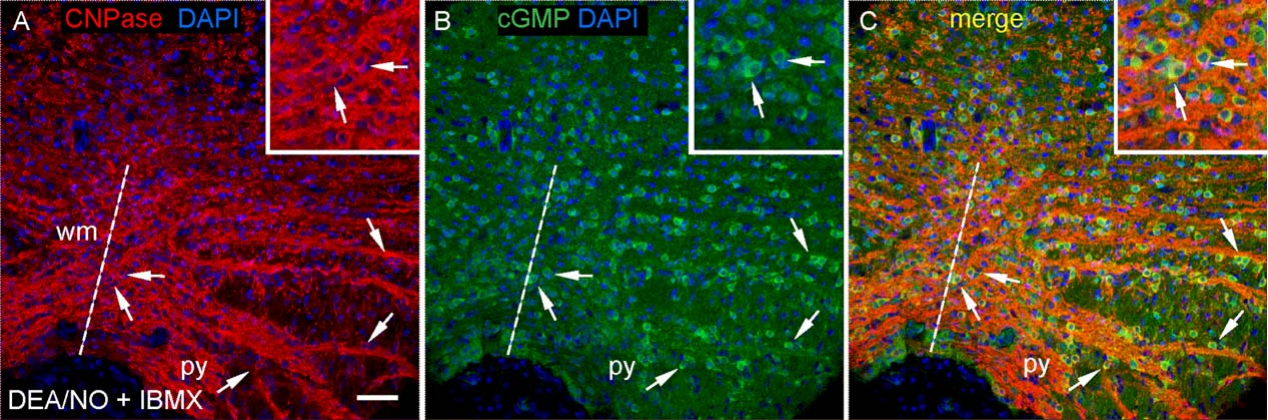
NO stimulates cGMP accumulation in oligodendrocytes in slices of 14-day-old rat brainstem. The slices were exposed to DEA/NO (100 μM, 5 min) in the presence of IBMX (1 mM). Images are from coronal sections at the level of the XII nerve, stained for (A) CNPase (red), (B) cGMP (green) and DAPI (blue). (C) overlay of A and B. Key: broken line, the mid line; py, pyramid; wm, white matter; arrows, oligodendrocytes. Insets are enlargements centred on the two arrowed cells near the midline. Scale (A) = 50 μm or 25 μm (insets). [Color figure can be viewed in the online issue, which is available at http://wileyonlinelibrary.com.]

As a partial test of the generality of the above findings, experiments were carried out on white matter-rich slices of brainstem from 14-day-old rats. Most of the oligodendrocytes accumulated cGMP in response to exogenous NO (this time donated by DEA/NO) and, conversely, most of the cGMP immunoreactivity was associated with oligodendrocytes (Fig. 6A–C).

### NO- and Natriuretic Peptide-Stimulated cGMP Accumulation in Rat Optic Nerve

We previously reported that, in the adult and immature rat optic nerve, macroglia (which include oligodendrocytes) did not appear to accumulate cGMP on exposure to NO, whereas axons became strongly cGMP-positive (Garthwaite et al., [Bibr b25],[Bibr b24]). Considering the divergent findings from both cerebellum and brainstem reported above, we sought to re-examine the responsiveness of optic nerve oligodendrocytes to NO and to test the possibility that their cGMP content may be regulated by natriuretic peptides instead.

**Figure 7:**
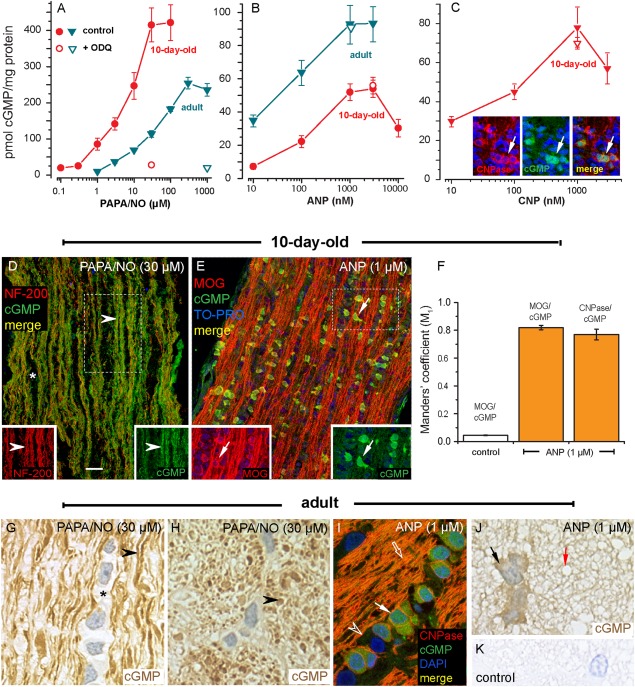
Differential targeting of axons and oligodendrocytes by NO and natriuretic peptides in rat optic nerve. (A–C) Concentration-cGMP response curves to PAPA/NO (A, 5 min), ANP (B, 10 min) and CNP (C, 10 min) in 10-day-old (red) or adult (blue) optic nerves in the absence (filled symbols) or presence (open symbols) of 10 μM ODQ (*n* = 5–9 nerves from 2–4 separate experiments). Insets in C: CNPase, cGMP and overlayed immunostaining in a nerve exposed to 1 μM CNP (10 min); arrows indicate a pair of the co-stained cells (yellow). (D, E) 10-day-old rat optic nerves exposed to PAPA/NO (30 μM, 5 min, D) or ANP (1 μM, 10 min, E) and stained for cGMP together with the axonal marker NF-200 (D) or the oligodendrocyte marker MOG (E). The main panels are overlays; fields of separate images from the boxed regions are in the insets. Key: arrowhead, axons; asterisk, band of macroglia; arrow, oligodendrocyte. (F) Manders' coefficient of colocalization between MOG and cGMP or CNPase and cGMP in the absence and presence of ANP (data from 3 nerves). (G–K) Semithin sections from adult optic nerve exposed to PAPA/NO (G, H) or ANP (I–K) in longitudinal (G, I) and transverse (H, J, K) planes. Panels G, H, J and K are immunoperoxidase staining for cGMP with haemalum counterstain. In G: asterisk, band of unstained macroglia. In G and H: arrowheads, cGMP-positive axons. In I: filled arrow, oligodendrocyte; arrowhead, presumed fibrous astrocyte; open arrow, myelinated axons. In J: black arrow, oligodendrocyte; red arrow, stained myelin (brown) surrounding an unstained axon. In panel K, the primary antibody was omitted. In all experiments, the nerves were incubated with IBMX (1 mM); microscopic images are representative of 4–6 nerves in 2–3 separate experiments. Nuclei (blue) were stained with DAPI (C, I) or TO-PRO (E). Scale bar (in D) = 20 μm (in D), 29 μm (E), 10.5 μm (G, H), 9 μm (I) and 7.5 μm (J, K). [Color figure can be viewed in the online issue, which is available at http://wileyonlinelibrary.com.]

When 10-day-old or adult rat optic nerves were incubated with either PAPA/NO or ANP (both in the presence of IBMX), cGMP levels became elevated in a concentration-dependent manner (Fig. 7A,B). The related peptide CNP also gave concentration-dependent increases of cGMP in 10-day-old rat nerves (Fig. 7C; adult nerves were not tested). Responses to NO, but not those to ANP or CNP, were inhibited by ODQ (Fig. 7A–C). Immunohistochemistry in 10-day-old nerves showed that PAPA/NO caused cGMP accumulation in axons whereas ANP and CNP appeared only to generate a response from oligodendrocytes (Fig. 7C–E). In accordance with this conclusion, after incubation with ANP, cGMP was strongly colocalized with either MOG or CNPase, as shown by quantitative analysis (Fig. 7F). The selective responsiveness of oligodendrocytes to ANP was also seen in the adult (Fig. 7I,J) in which additional immunostaining of presumed myelin sheaths could also be detected (Fig. 7J,K). In contrast, as in the 10-day-old optic nerves, PAPA/NO-evoked cGMP accumulation appeared in axons but not in oligodendrocytes (Fig. 7G) nor in myelin sheaths (Fig. 7H).

**Figure 8:**
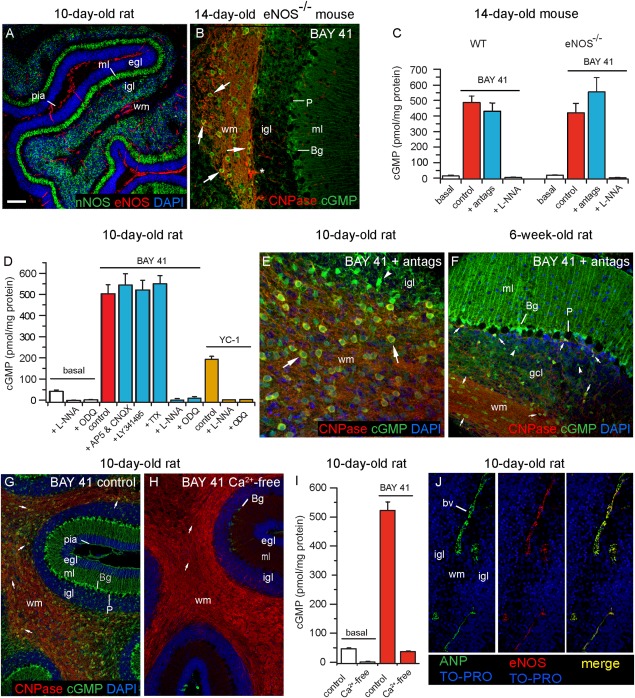
Sources of endogenous agonists acting to increase cGMP in oligodendrocytes in cerebellum. (A) Section from 10-day-old rat cerebellar slice stained for eNOS (red), nNOS (green), and nuclei (DAPI, blue). (B) Section of a slice from a 14-day-old eNOS^−/−^ mouse incubated with BAY 41-2272 (3 μM; 5 min) and stained for CNPase (red) and cGMP (green), showing cGMP accumulation in oligodendrocytes (examples arrowed). Asterisk indicates non-specific staining of blood vessel (staining persisted after omission of primary antibody). (C) cGMP radioimmunoassay measurements in slices of wild-type (WT) and eNOS^−/−^ mouse cerebellum showing responses to BAY41-2272 (red bars) in the absence and presence of L-nitroarginine (L-NNA, 100 μM) and of a mixture of antagonists (“antags,” blue bars) of ionotropic glutamate receptors (100 μM AP5 plus 100 μM CNQX) and of Na^+^ channels (1 μM TTX). Data are from 6–9 slices in three separate experiments; the population means were not significantly different from each other (*P* > 0.05 by one-way ANOVA). (D) Cerebellar slices from 10-day-old rats exposed for 5 min to BAY41-2272 (BAY 41; 3 μM, left columns) or YC-1 (50 μM, right columns) under the indicated conditions. The concentrations of AP5, CNQX, TTX and L-NNA were as given above; ODQ was at 10 μM and the general metabotropic glutamate receptor blocker LY341495 was at 100 μM; *n* = 4-12 (2–5 separate experiments). (E, F) Sections of 10-day-old (E) and 6-week-old (F) rat cerebellar slices exposed to BAY 41-2272 (3 μM, 5 min) in the presence of a mixture of AP5, CNQX and TTX (“antags”), immunostained for cGMP (green) and CNPase (red). (G, H) Immunostained sections of 10-day-old rat cerebellar slices exposed to BAY 41-2272 (5 μM, 5 min) in the presence (G) or absence (H) of Ca^2+^. (I) cGMP levels in the absence (open columns) and presence (red columns) of BAY 41-2272 (3 μM; 5 min) under control and Ca^2+^-free conditions (as indicated); *n* = 7–9 (three separate experiments). IBMX (1 mM) was present throughout. (J) Images from a 10-day-old rat cerebellar slice immunostained for ANP and/or eNOS as indicated; nuclei are stained with TO-PRO (blue). In the micrographs, arrows point to oligodendrocytes and arrowheads signify putative astrocytes. Key: egl, external granule cell layer; igl, internal granule cell layer; gcl, granule cell layer; ml, molecular layer; Bg, Bergmann glia; wm, white matter. Scale bar (in A) = 100 μm (A); 35 μm (B): 26 μm (E); 50 μm (F, J) and 80 μm (G, H). All images are representative of four slices in two separate experiments. Histogram results (C, D, I) are means ± SEM. [Color figure can be viewed in the online issue, which is available at http://wileyonlinelibrary.com.]

Optic nerves from animals as young as 1-day-old responded to PAPA/NO or BAY 41-2272 with axonal cGMP accumulation whereas, with ANP, only a few CNPase-positive oligodendrocytes in the 7-day-old tissue exhibited faint cGMP immunostaining (2 ± 0.33%; *n* = 3 nerves from different animals), with no significant variation with position along the nerve (*P* > 0.05 by ANOVA). At this age, oligodendrocytes did not stain detectably for MOG and there was very limited MBP immunostaining. In the 3-day-old tissue, CNPase-positive cells were observed only in the chiasm but no cGMP immunostaining of these cells (or of the abundant NG2-positive cells) after exposure to ANP was observed (Supp. Info. Fig. S2).

### Sources of Endogenous Agonists Generating cGMP in Cerebellar Oligodendrocytes

The two constitutive NO synthases, eNOS and nNOS, showed a differential distribution in the developing rat cerebellum, eNOS being confined to blood vessels and nNOS being expressed in granule cells in the internal granule cell layer and in the molecular layer (Fig. 8A), where it is primarily located in inhibitory interneurones (Bredt et al., 1990; Wood et al., 2011). As NO synthase inhibitors able to distinguish between the two isoforms experimentally in intact cells and tissues are still lacking (Pigott et al., 2013), cGMP immunostaining was applied to cerebellar slices from wild-type mice and mice deficient in eNOS. On exposure to BAY 41-2272, the staining was indistinguishable, with cGMP being found in oligodendrocytes as well as in Bergmann glia and other presumed astrocytes (Fig. 8B), as found in the rat tissue. cGMP levels in the presence of BAY 41-2272 were not significantly altered in slices from eNOS knockouts but, as in the wild-type tissue, they were sensitive to inhibition by L-nitroarginine (Fig. 8C), confirming that they were sustained by NO synthase activity.

The lack of obvious participation of eNOS in the basal NO tone of the tissue differs from findings made on optic nerve (Garthwaite et al., 2006). Given this apparent disparity, the previous result was reexamined using optic nerves from 11-day-old mice. Nerves from wild-type mice exposed to BAY 41-2272 (3 µM, 5 min, in the presence of 1 mM IBMX) produced 135 ± 18 pmol cGMP/mg protein whereas nerves from eNOS knockouts gave only 13 ± 5 pmol cGMP/mg protein (*n* = 6 nerves from two experiments). This result compares well with that reported previously (Garthwaite et al., 2006), indicating that the tissue differences with respect to the participation of eNOS in basal NO levels are real.

The findings with eNOS-knockout mice suggest that nNOS is the main candidate enzyme responsible for basal NO in the cerebellum. nNOS typically becomes active in response to stimulation of NMDA receptors, although non-NMDA receptors could, in principle, also participate (Southam et al., 1991). When slices were exposed to BAY 41-2272, cGMP levels in either wild-type or eNOS-knockout tissues were unaffected by a mixture of antagonists for NMDA and non-NMDA receptors (AP5 + CNQX), together with a blocker of voltage-gated Na^+^ channels (TTX) to inhibit action potential-dependent network activity (Fig. 8C). Similarly, in slices from the 10-day-old rat exposed to BAY 41-2272, cGMP levels were unaffected by AP5 plus CNQX, or TTX, or an inhibitor of metabotropic glutamate receptors (LY 341495) at a concentration (100 µM) high enough to act without subtype selectively (Kingston et al., 1998) (Fig. 8D). In accordance with these results, oligodendrocyte cGMP immunostaining persisted in slices of both the 10-day-old and adult rat cerebellum exposed to BAY 41-2272 in the combined presence of AP5, CNQX, and TTX (Fig. 8E,F).

As GABA may raise cerebellar cGMP (Ferrendelli et al., 1974), the GABA_A_ antagonist bicuculline (30 µM) was also tested but found to have no significant effect: cGMP levels were 341 ± 36 pmol/mg protein in the control slices exposed to BAY 41-2272 (1 µM) and 241 ± 25 with bicuculline additionally included (*n* = 4–5; *P* > 0.05). As expected for nNOS, the cGMP immunostaining and elevation in cGMP levels were virtually abolished when the slices were incubated under Ca^2+^-free conditions (Fig. 8G–I), a result that also eliminates from consideration the Ca^2+^-independent inducible NO synthase isoform (Cho et al., 1992).

Possible endogenous sources of ANP in the 10-day-old rat cerebellum were also investigated. ANP immunostaining coincided with eNOS (Fig. 8J), implying that the peptide is present and is mainly located in blood vessels in the developing tissue. In the 10-day-old rat optic nerve, ANP immunostaining also colocalized with eNOS and the distribution of CNP immunolabeling likewise suggested that this peptide is mainly present in blood vessels (Supp. Info. Fig. S3).

### Oligodendrocytes as NO Targets in Cortical Cultures: Developmental Characterization

We speculated from the ontogenesis of cGMP accumulation in cerebellar oligodendrocytes in response to NO and ANP that cGMP might have a physiological role in oligodendrocyte maturation. Experiments were carried out to determine the suitability of cultures derived from embryonic mouse cerebral cortex for this purpose.

**Figure 9:**
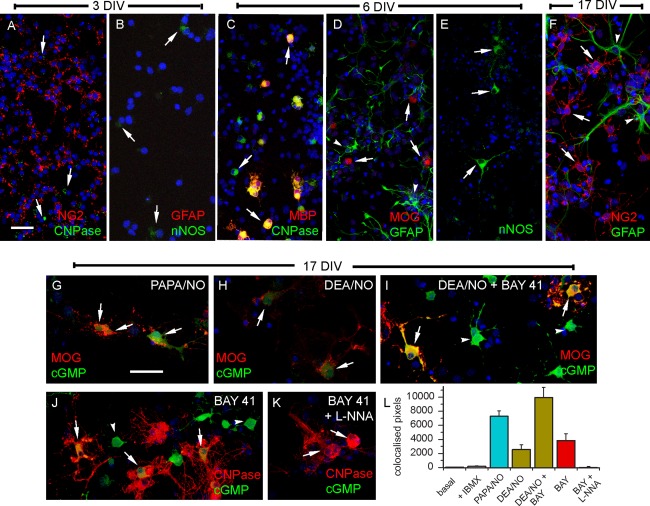
Differentiation of oligodendrocytes in cortical cultures and their responsiveness to exogenous and endogenous NO. (A–F) Cultures at 3 DIV (A, B), 6 DIV (C–E), and 17 DIV (F) immunostained for the indicated markers. Arrows point to examples of CNPase-positive oligodendrocytes (A), nNOS-positive cells (B, E), CNPase-positive and/or MBP-positive oligodendrocytes (C), MOG-positive oligodendrocytes (D), and NG2-positive cells (F); arrowheads, examples of GFAP-positive astrocytes (D, F). Scale bar (in A) = 40 μm (for A, C–F) and 29 μm (B). (G–K) cGMP immunostaining (green) in oligodendrocyes (red) immunolabeled with CNPase or MOG at 17 DIV following exposure to PAPA/NO (30 μM, 2 min; G), DEA/NO (100 nM, 2 min; H, I) and/or BAY 41-2272 (1 μM, 7 min; I–K) in the absence (J) or presence (K) of L-nitroarginine (L-NNA, 100 μM), all in the presence of IBMX (1 mM). Arrows, cGMP-positive (G–J) or negative (K) oligodendrocytes; arrowheads, examples of cGMP-positive putative astrocytes (I, J). Scale bar (G) = 40 μm (for G–K). (L) Numbers of pixels containing both red (CNPase) and green (cGMP) signals derived from z-stacks (1 μm) of 0.17 mm^2^ images of cultures under the indicated conditions (concentrations and exposure periods as above); data are means ± SEM from three coverslips in two experiments. [Color figure can be viewed in the online issue, which is available at http://wileyonlinelibrary.com.]

Early on (3 days after seeding) many cells stained positively for NG2, a marker of oligodendrocyte precursor cells, with only the occasional cell showing faint staining for CNPase, an early-appearing marker of oligodendrocyte differentiation (Fig. 9A). There were also a few cells that stained faintly for nNOS, while none stained for the astrocytic marker GFAP (Fig. 9B). At 6 days *in vitro* (DIV), distinct CNPase-positive cells appeared, some of which co-stained for MBP, a marker for more mature oligodendrocytes (Fig. 9C). At this stage, some cells expressed MOG, a marker of mature oligodendrocytes (Fig. 9D) and GFAP-positive cells were easily recognizable (Fig. 9D). nNOS-positive cells and their processes stained robustly (Fig. 9E), although they were small in number, accounting for 3 ± 0.7% of the total cell population (from four coverslips in two experiments).

At 17 DIV, NG2-positive cells with preoligodendrocyte morphology were still present (Fig. 9F), together with cells staining for CNPase, MOG, and MBP (see below). Cultures at this age were selected to test for responsiveness to exogenous and endogenous NO. Two different NO donors, PAPA/NO (30 µM, 2 min) and DEA/NO (100 nM, 2 min) elicited cGMP immunostaining in MOG-positive (Fig. 9G,H) and CNPase-positive oligodendrocytes as shown by quantitative image analysis (Fig. 9L). Incubation with BAY 41-2272 (1 µM) also resulted in cGMP accumulation in CNPase-positive oligodendrocytes (Fig. 9J,L), a response that was blocked by L-nitroarginine (Fig. 9K,L). The combination of DEA/NO and BAY 41-2272 increased oligodendrocyte cGMP more than each stimulant alone (Fig. 9I,L).

### Effects of the NO-cGMP Pathway on Oligodendrocyte Arborization

**Figure 10:**
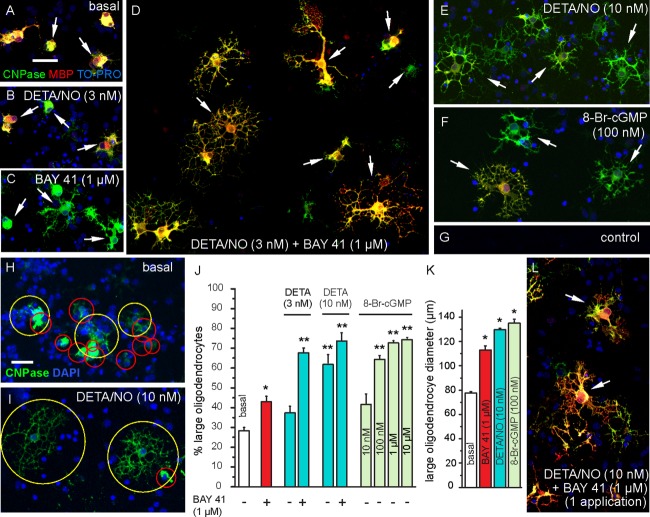
Effect of NO-cGMP signaling on the morphological differentiation of cultured oligodendrocytes. Cultures were exposed to the stated conditions from 3 DIV (A–K) or 6 DIV (L) and fixed at 8 DIV. Oligodendrocytes (examples arrowed) were immunolabeled for CNPase and MBP (A, B, D–F, L) or for CNPase alone (C, H, I); nuclei were stained with TO-PRO or DAPI as indicated. Primary antibodies were omitted in the control (G). The method used to quantify the spread of oligodendrocytes is illustrated in H and I, where the red circles demarcate territories of 70 μm or less in diameter and yellow circles, territories above this value. The histograms show the percentage of cells >70 μm in diameter (J) and their mean diameter (K) under selected conditions; data are means ± SEM from five areas (each 1 mm^2^) per coverslip and 3–8 coverslips in 2–3 experiments. In J, *significantly different from basal (*P* < 0.05); ** significantly different from basal and from BAY 41-2272 alone (*P* < 0.05) but not from each other; in K, *significantly different from basal (*P* < 0.05) but not from each other (one-way ANOVA). Micrographs are representative illustrations from the same experiments. Key: BAY 41, BAY 41-2272; DETA (in J), DETA/NO. Scale bar (in A) = 40 μm (for A–G, L); scale bar (in H) = 50 μm (for H, I). [Color figure can be viewed in the online issue, which is available at http://wileyonlinelibrary.com.]

Having established that the cultured cortical oligodendrocytes resembled their counterparts in cerebellar and brainstem slices in being NO targets, experiments were carried out to determine if this signaling pathway affected their morphological development. For this purpose, the cultures were immunostained for CNPase and MBP. Under all conditions tested, some cells at 8 DIV stained only for CNPase but more of them stained for both markers (Fig. 10A,B,D–F,L), signifying a more advanced maturational state. The majority of oligodendrocytes in untreated cultures had limited arborization (Fig. 10A).

To quantify the extent of arborization, we adopted a simple yardstick (see Materials and Methods) whereby a circle was used to circumscribe the territory of each oligodendrocyte (see Fig. 10H,I) and the numbers of cells whose territorial diameter was greater than a particular value, arbitrarily chosen as 70 µm, were counted (Fig. 10J). The controls had 28 ± 2% of oligodendrocytes meeting this criterion (from eight coverslips in four experiments). In an attempt to emulate endogenous NO production, cultures were exposed from 3 DIV to 8 DIV (when they were fixed) to low concentrations of DETA/NO (3 and 10 nM), a donor that has a long half-life (20 h) and so releases NO very slowly (initially 3 and 10 pM per min, respectively). With 3 nM DETA/NO, no significant morphological effects were seen (Fig. 10B,J). At 10 nM DETA/NO, however, there was a marked increase in the spread of the oligodendrocyte arbor (Fig. 10E,I) that was reflected in a 2.2-fold increase in the number of cells with territorial diameters exceeding 70 µm (Fig. 10J). This increase appeared to represent a general shift in the population to larger sizes because the mean territorial diameter of cells >70 µm increased by 67% (Fig. 10K).

BAY 41-2272 (1 μM) on its own had a relatively smaller, but significant, effect on the proportion of larger oligodendrocytes (Fig. 10C,J) and also increased their mean size (Fig. 10K). When BAY 41-2272 was combined with the normally subthreshold concentration of DETA/NO (3 nM), the percentage of large oligodendrocytes became similar to that found at the higher DETA/NO concentration (Fig. 10D,J). Adding BAY 41-2272 to the higher DETA/NO concentration (10 nM) gave no significant further increase in oligodendrocyte spread (Fig. 10J), suggesting that this DETA/NO concentration on its own was maximal. The ability of BAY 41-2272 to enhance the response to the low DETA/NO concentration provides good evidence that NO-activated guanylyl cyclase is the downstream effector. In aiming to further test this possibility, ODQ was initially used but it was found to be toxic to the cells during prolonged exposure, in common with findings made on other cultures (Haramis et al., 2008). As an alternative approach, the membrane-permeating cGMP analogue, 8-bromo-cGMP was applied (from 3 DIV onwards). At 10 nM, 8-bromo-cGMP had no significant effect but higher concentrations led to a progressively increasing proportion of large oligodendrocytes (Fig. 10F), the half-maximal effect occurring at <100 nM and a maximum change (at 1–10 µM) that was comparable with that found after exposure to 10 nM DETA/NO (Fig. 10J). The mean size of the larger oligodendrocyte population in the presence of 100 nM 8-Br-cGMP increased by an amount similar to that found with 10 nM DETA/NO (Fig. 10K).

Although the duration of the exposures used above was for several days, the combination of DETA/NO (10 nM) plus BAY 41-2272 added at 6 DIV was found to have a similar effect to when it was applied 3 days earlier (Fig. 10L), indicating that the more prolonged (6 days) exposure is not required to bring about the observed morphological development of oligodendrocytes.

## Discussion

The results indicate that oligodendrocytes in the rat cerebellum are physiological targets for neuronally-derived NO from early postnatal development into adulthood, that responsiveness to NO (exhibited as cGMP accumulation) may be a widespread property of oligodendrocytes, that ANP or CNP can provide alternative signals for oligodendrocyte cGMP accumulation, and that a downstream consequence of this signaling pathway is enhanced morphological development.

There had previously been scant evidence for considering oligodendrocytes as recipients of physiological NO signals. In the developing and adult cerebellum, use of cGMP immunocytochemistry had highlighted astrocytes in white and grey matter (de Vente et al., 1990) but no attempt was made to determine if oligodendrocytes might also be NO- or ANP-responsive. In the only directly relevant study, it was found that NO raised cGMP in oligodendrocytes in the corpus callosum of 2-week-old rats and that this responsiveness declined with age thereafter (Tanaka et al., 1997). The same study noted that ANP was unable to elicit detectable cGMP accumulation in oligodendrocytes at the age when they displayed NO-responsiveness. In a separate investigation on rat whole-brain spheroid cultures, occasional oligodendrocytes were found to accumulate cGMP in response to sodium nitroprusside, which releases NO (amongst other products), or to NMDA, but not to ANP (Teunissen et al., 2000). Conversely, prolonged exposure to ANP elevated cGMP in an oligodendrocyte-like cell line (Yoshioka et al., 2000) and myelin purified from rat brainstem was reported to contain guanylyl cyclase activity that was insensitive to sodium nitroprusside, suggesting a natriuretic peptide receptor-associated guanylyl cyclase (Chakraborty and Ledeen, 1993). Together, these previous studies are consistent with oligodendrocytes having the capacity to generate cGMP on stimulation by NO and/or ANP.

In the rat cerebellar slices, both NO and ANP raised cGMP in oligodendrocytes from around postnatal day 8 through to adulthood, findings that differ from those reported using the corpus callosum with respect to both age-dependence and sensitivity to ANP (Tanaka et al., 1997), pointing to a possible heterogeneity amongst oligodendrocytes in different brain regions. Oligodendrocytes in 10-day-old rat hippocampal slices failed to display cGMP immunostaining after exposure to exogenous NO plus BAY 41-2272 (Bartus et al., 2013) but these experiments were conducted in the presence of a selective inhibitor of phosphodiesterase-2, an enzyme that may not feature prominently in oligodendrocyte cGMP breakdown, so that cGMP (if generated) may have remained below detection. In the present experiments, oligodendrocyte cGMP immunostaining in response to NO was found not only in slices of cerebellum but also in slices of brainstem and in cultures derived from the cerebral cortex, indicating that it is likely to be a common property of this cell type. An exception was in the rodent optic nerve where axons, but not astrocytes or oligodendrocytes, exhibited NO-sensitivity, a result that agrees with previous findings (Garthwaite et al., [Bibr b25],[Bibr b24]). Instead, beyond 7 days of age, rat optic nerve oligodendrocytes responded to ANP or CNP, indicating that these particular oligodendrocytes preferentially express transmembrane guanylyl cyclase-coupled receptors. Given the similar potencies of ANP and CNP, the optic nerve oligodendrocytes presumably contain both subtypes of receptors (NPR-A and -B) whereas cerebellar cGMP in the guinea-pig is much more sensitive to ANP than to CNP (Hernandez et al., 1994), suggesting that NPR-A receptors dominate.

While being a valuable technique, cGMP immunohistochemistry suffers the limitation of not being sensitive to the submicromolar cGMP concentrations that are likely to be important physiologically (Bartus et al., 2013). The use of allosteric enhancers of NO-activated guanylyl cyclase such as BAY 41-2272 help address this deficiency by boosting the guanylyl cyclase activity sustained by low NO concentrations sufficiently to elevate cGMP into the detectable range. In this respect, physiological NO signals in the brain are likely to be in the picomolar-nanomolar range, which is at the base of the NO concentration-response curve for its receptors in cells (Hall and Garthwaite, 2009). By exploiting allosteric enhancers of this activity, our experiments showed that endogenously produced NO was able to access oligodendrocytes in cerebellar slices. Unlike in optic nerve (Garthwaite et al., 2006) the experiments all pointed to nNOS (rather than eNOS) being the prominent isoform governing the basal NO tone in the cerebellum. Here, granule cells are the nNOS-expressing neurones physically closest to oligodendrocytes and so they represent the most likely cellular origins of the NO signals (oligodendrocytes themselves apparently do not contain NO synthase; Keilhoff et al., 1998). Although NMDA receptor stimulation is an effective stimulus for NO generation by granule cells (Wood et al., 2011), the nNOS activity that is transduced into oligodendrocyte cGMP accumulation was independent of ongoing glutamate receptor stimulation, suggesting that it is maintained by an atypical mechanism. Possibilities include stimulation by Ca^2+^ entering through voltage-dependent Ca^2+^-channels or, by analogy with eNOS (Fulton et al., 2001), nNOS becoming active at resting Ca^2+^ concentrations after being phosphorylated.

Concerning endogenous sources of ANP, a previous study failed to detect the peptide in the cerebellum by radioimmunoassay or immunohistochemistry (Kawata et al., 1985) whereas, elsewhere in the brain, ANP has been found mainly in neurones or astrocytes (Cao and Yang, 2008). Our finding that ANP is associated with blood vessels in the developing cerebellum, as are both ANP and CNP in the developing optic nerve, signifies that there might be a local peptidergic signaling pathway able to raise cGMP in oligodendrocytes.

Myelination in the rat cerebellum begins in the central white matter at around postnatal day 6 and proceeds along the axial core of the lobules to appear in the internal granule cell layer 4–6 days later (Gianola et al., 2003; Reynolds and Wilkin, 1988). The development of oligodendrocyte NO-sensitivity, being undetectable at postnatal day 3 and prominent at day 8, would be consistent with a role in this process. Tests of this possibility using cortical cultures showed a striking effect of NO or cGMP on oligodendrocyte morphology, exhibited as a shift to a greatly enhanced arborization. Based on a previous classification, the cells shifted from a predominantly type I/II to a type III/IV morphology and, in this respect, closely resembled the changes of cultured cortical oligodendrocytes recorded on exposure to adenosine (Stevens et al., 2002). From calculations (see Materials and Methods), the NO concentration produced by the maximally-effective donor concentration in these experiments (10 nM DETA/NO) peaks at 88 pM after 50 min and then declines to 40 pM after 24 h. These NO concentrations are similar to the ones recorded from the internal granule cell layer in 10-day-old rat cerebellar slices following brief (45 s) stimulation with a half-maximally-effective concentration of NMDA (Wood et al., 2011). The donor concentration that was inactive on its own (3 nM) is predicted to yield NO concentrations of 12–27 pM over 24 h, concentrations that may be below detection (in the absence of BAY 41-2272), particularly in cells with high cGMP phosphodiesterase activity (Batchelor et al., 2010). That the effect of NO was enhanced by BAY 41-2272 and was mimicked by 8-bromo-cGMP at the submicromolar concentrations relevant to activation of cGMP-dependent protein kinases (Pohler et al., 1995) supports guanylyl cyclase activation being the key pathway engaged by NO to promote oligodendrocyte maturation.

Transduction of cGMP signals commonly occurs through cGMP-dependent protein kinases (PKG), of which there are two main types, PKGI and PKGII. PKGI has a restricted distribution in the central nervous system whereas PKGII is widespread and is present in putative oligodendrocytes (de Vente et al., 2001) and an oligodendrocyte-like cell line (Yoshioka et al., 2000), making this isoform the probable downstream target of NO or ANP. The subsequent steps leading to the morphological development of oligodendrocytes remain unexplored, but a number of possibilities exist. One substrate for PKGII is glycogen synthase kinase-3β (GSK-3β), with the resulting phosphorylation leading to inhibition of enzyme activity and, in chondrocytes, the hypertrophic differentiation needed for normal development of the skeleton (Kawasaki et al., 2008). Likewise, in oligodendrocytes, GSK-3β negatively influences differentiation and myelination and its pharmacological inhibition has the opposite effect and promotes remyelination in focal demyelinated lesions *in vivo* (Azim and Butt, 2011). In the peripheral nervous system, inhibition of GSK-3β similarly enhances Schwann cell differentiation and axon myelination (Makoukji et al., 2012; Ogata et al., 2004). Hence, inhibition of GSK-3β by PGKII-mediated phosphorylation could plausibly account for our observations. Alternatively, a putative PKG substrate in oligodendrocytes is the transcription factor Olig1 which, on phosphorylation, locates in the cytoplasm to evoke morphological changes similar to those described here in response to NO or 8-bromo-cGMP (Niu et al., 2012). Also PKGII is a potent regulator of gene expression in glioma cells (Gudi et al., 1999), particularly under conditions of raised intracellular Ca^2+^, although such an action in oligodendrocytes has not been tested.

Examination of our conclusions *in vivo* will be an important future step, but some pertinent evidence already exists. In rodents, inhaling air supplemented with NO (5 or 20 ppm) during the first postnatal week was found to enhance myelination in the corpus callosum in the 7- or 14-day-old animal, whereas inhibition of NO synthase activity over the same period significantly reduced myelin density, an effect that could be abrogated by administering inhaled NO (Olivier et al., 2010). Also, mice deficient in nNOS exhibited a delay in remyelination following chemical demyelination (Linares et al., 2006). These findings provide partial support for a role of the NO/cGMP pathway in oligodendrocyte maturation *in vivo*.

The development of oligodendrocytes into myelinating cells involves a complex interplay between intrinsic and extrinsic factors. Amongst the latter, axon-derived ATP (giving rise to extracellular adenosine) and glutamate have been implicated as local drivers of oligodendrocyte maturation and axon myelination (Emery, 2010; Zuchero and Barres, 2013). Our results add NO to the list of putative neuronally-derived factors but, in the cerebellum at least, the axons undergoing myelination do not contain detectable NO synthase, making a localized mechanism unlikely. Rather, NO produced in the nearby granule cells may be acting more as a volume transmitter to enable the myelination of afferent and efferent axons to keep pace with the activity of the cerebellum as it develops. In the corpus callosum, intrinsic nNOS neurones (Barbaresi et al., 2014; Rockland and Nayyar, 2012) may perform this role. By analogy with other brain areas (Gibson et al., 2014; Young et al., 2013), the persistence of this signaling pathway into adulthood in the cerebellum could be significant for the adaptive changes in myelination that may be of importance for adult motor learning.
