# Supplementary material for: Nitric oxide targets oligodendrocytes and promotes their morphological differentiation
Source: Glia. 2014 Oct 18;63(3):383–99. doi: 10.1002/glia.22759 (PMC4309495; doi:10.1002/glia.22759)
Supplement: Supplementary file 1 [file glia0063-0383-sd1.doc]

**Supporting Information**

**Supporting Figure Legends**


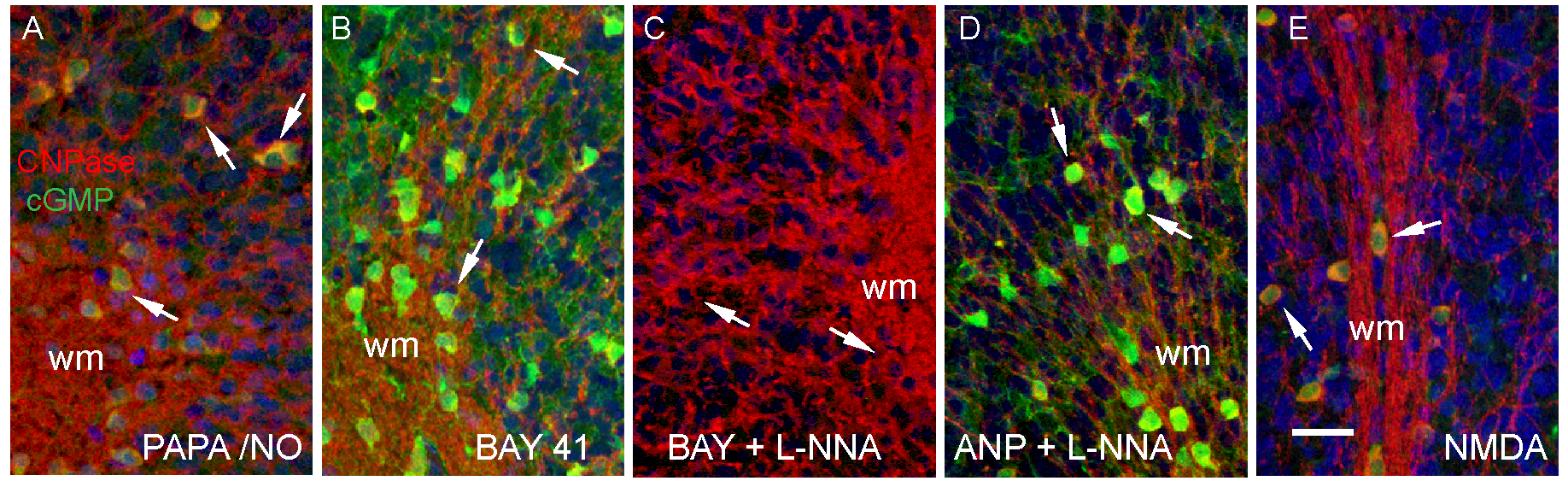


Figure S1. Enlargements from each of the panels in Fig. 5, centred on the white matter. Scale bar (E) = 25 µm.


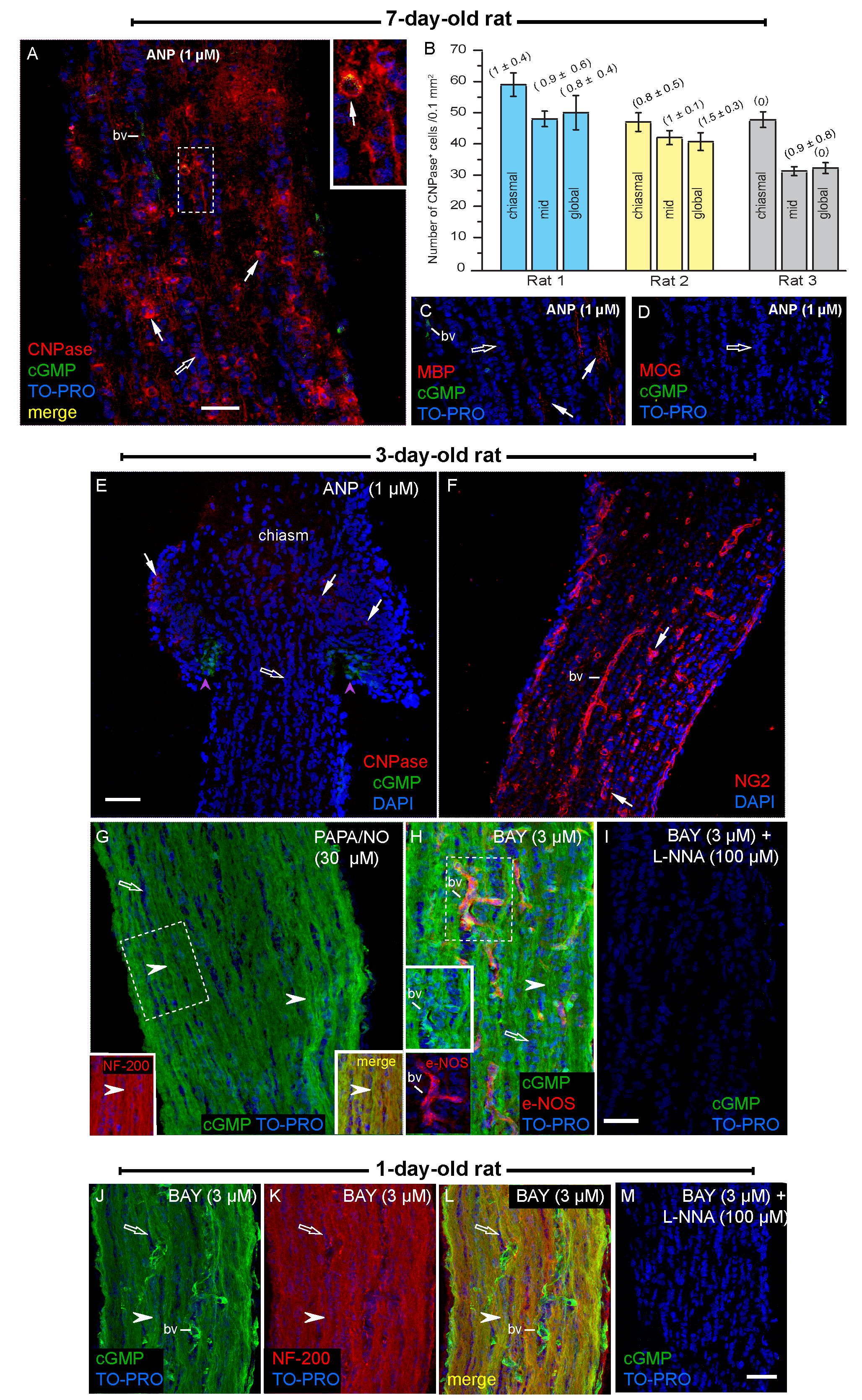


Figure S2**.** Agonist-evoked cGMP accumulation in rat optic nerve oligodendrocytes and axons during early postnatal development. **A-D**: 7-day-old tissue. Many CNPase-positive oligodendrocytes in different stages of differentiation are present (**A**) but there is little cGMP immunostaining in response to ANP (1 µM, 10 min); a rare example of a co-stained cell is shown in the inset. In contrast, blood vessels (bv) stained positively for cGMP. The histogram (**B**) quantifies the density of CNPase-positive oligodendrocytes in 3 sub-regions of nerves from 3 different rats, measured in 400 x 400 µm fields (data normalized to 0.1 mm^2^); values in parentheses are the numbers of co-stained cells within the same areas. Data are means ± SEM from 3 sections taken from each nerve sub-region, and one nerve per animal. Occasional MBP staining in presumed oligodendrocyte processes was seen (**C**) but no MOG staining was detected (**D**). Scale bar (in **A)** = 40 µm (**A**) and 21 µm (**C,** **D**). **E-I**: 3-day-old tissue. Nerves incubated with ANP (1 µM, 10 min) showed CNPase-positive cells (red) restricted to the chiasmatic end (**E**), in accordance with oligodendrocyte differentiation following a chiasm-to-globe gradient (Colello et al., 1995). No cells in the optic nerve stained for cGMP (green) but unidentified cGMP-positive cells were found within the meningeal sheath surrounding the chiasm (pink arrowheads). NG2-positive cells, which include oligodendrocyte precursor cells, were seen throughout the nerve (**F**); blood vessels (bv) were also stained. Exposure of 3-day-old nerves to PAPA/NO (30 µM, 5 min) generated robust cGMP immunostaining (green) that was located in axons, as verified by co-staining with NF-200 (red, inset) and overlaying the images (yellow, inset). Similar axonal cGMP was found after exposure to BAY 41-2272, (3 μM, 5 min), an allosteric activator of NO-stimulated guanylyl cyclase; this response was blocked by the NO synthase inhibitor L-nitroarginine (L-NNA; **H,I**), confirming mediation by endogenous NO. Co-staining for eNOS (red, **H**) identifies blood vessels (bv) as the likely endogenous NO source, as was found in nerves from older animals (Garthwaite et al., 2006). Scale bar (in **E**) = 50 µm (applies to **E** and **F**); scale bar (in **I**) = 40 µm (applies to **G-I**). **J-M**: 1-day-old tissue. BAY 41-2272 retained its ability to evoke cGMP accumulation in axons (**J-L**) in an L-nitroarginine-sensitive manner (**M**), indicating that endogenous NO targeted axons even at this early age. Scale bar (in **M**) = 40 µm (applies to **J-M**). All sections are cut in the longitudinal plane and nuclei (blue) are stained with TO-PRO (**A,C,D,G-M**) or DAPI (**E,F**). Insets are of the boxed regions in the main images. Key: filled arrows, CNPase-positive oligodendrocytes or, in **F**, NG2-positive putative oligodendrocyte precursors; open arrows, row of macroglia; arrowheads, axons. All images are representative examples of 3-5 nerves studied in 2 experiments.


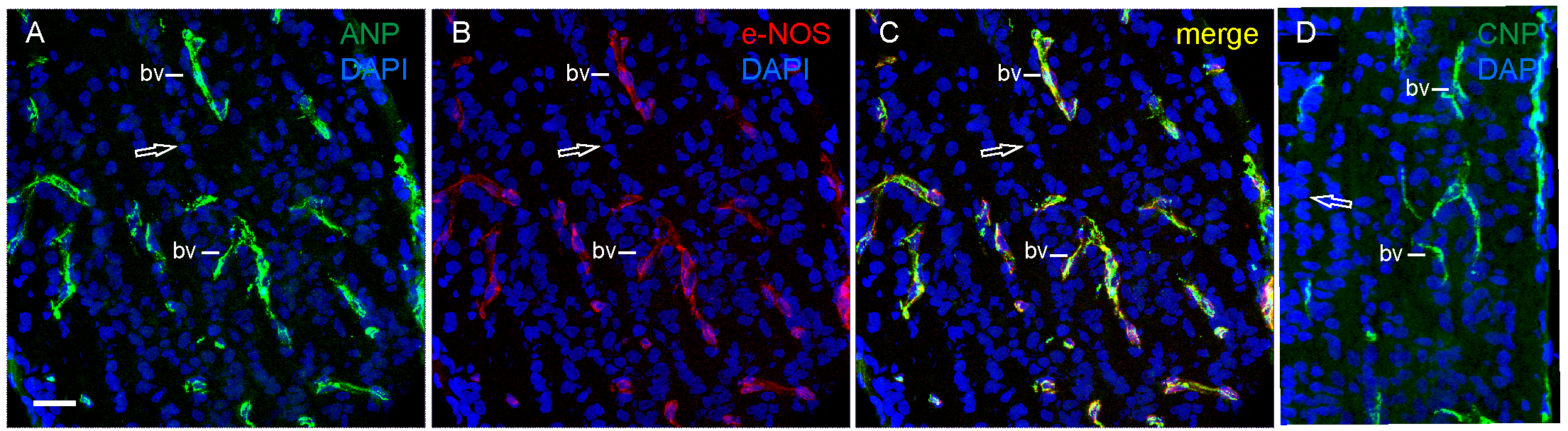


Figure S3. The location of atriopeptins in 10-day-old rat optic nerve. ANP immunoreactivity (**A**) is seen to co-localize with eNOS (**B,C**), indicating that the peptide is present in blood vessels. Immunoreactivity for CNP (**D**) displays the same pattern, suggesting the same location. Key: arrows, nuclei of macroglia (stained blue with DAPI); bv, blood vessel. Scale bar (in **A**) = 25 μm for all panels. Images are representative of 3 nerves.

Supporting References

Colello RJ, Devey LR, Imperato E, Pott U. 1995. The chronology of oligodendrocyte differentiation in the rat optic nerve: evidence for a signaling step initiating myelination in the CNS. J Neurosci 15: 7665-7672.

Garthwaite G, Bartus K, Malcolm D, Goodwin DA, Kollb-Sielecka M, Dooldeniya C, Garthwaite J. 2006. Signaling from blood vessels to CNS axons through nitric oxide. J Neurosci 26: 7730-7740.
